# Glutamate alleviates muscle protein loss by modulating TLR4, NODs, Akt/FOXO and mTOR signaling pathways in LPS-challenged piglets

**DOI:** 10.1371/journal.pone.0182246

**Published:** 2017-08-04

**Authors:** Ping Kang, Xiuying Wang, Huanting Wu, Huiling Zhu, Yongqing Hou, Longmei Wang, Yulan Liu

**Affiliations:** Hubei Collaborative Innovation Center for Animal Nutrition and Feed Safety, Hubei Key Laboratory of Animal Nutrition and Feed Science, Wuhan Polytechnic University, Wuhan, People’s Republic of China; Universidade de Sao Paulo, BRAZIL

## Abstract

The experiment was conducted to study the effect of the glutamate (Glu) on muscle protein loss through toll-like receptor 4 (TLR4), nucleotide-binding oligomerization domain proteins (NODs), Akt/Forkhead Box O (Akt/FOXO) and mammalian target of rapamycin (mTOR) signaling pathways in LPS-challenged piglets. Twenty-four weaned piglets were assigned into four treatments: (1) Control; (2) LPS+0% Glu; (3) LPS + 1.0% Glu; (4) LPS + 2.0% Glu. The experiment was lasted for 28 days. On d 28, the piglets in the LPS challenged groups were injected with LPS on 100 μg/kg body weight (BW), and the piglets in the control group were injected with the same volume of 0.9% NaCl solution. After 4 h LPS or saline injection, the piglets were slaughtered and the muscle samples were collected. Glu supplementation increased the protein/DNA ratio in gastrocnemius muscle, and the protein content in longissimus dorsi (LD) muscle after LPS challenge (*P*<0.05). In addition, Glu supplementation decreased TLR4, IL-1 receptor-associated kinase (IRAK) 1, receptor-interacting serine/threonine-protein kinase (RIPK) 2, and nuclear factor-κB (NF-κB) mRNA expression in gastrocnemius muscle (*P*<0.05), MyD88 mRNA expression in LD muscle, and FOXO1 mRNA expression in LD muscle (*P*<0.05). Moreover, Glu supplementation increased p-Akt/t-Akt ratio (*P*<0.05) in gastrocnemius muscle, and p-4EBP1/t-4EBP1 ratio in both gastrocnemius and LD muscles (*P*<0.05). Glu supplementation in the piglets’ diets might be an effective strategy to alleviate LPS-induced muscle protein loss, which might be due to suppressing the mRNA expression of TLR4 and NODs signaling-related genes, and modulating Akt/FOXO and mTOR signaling pathways.

## Introduction

Toll-like receptor (TLR) 4 as a key component of the innate immunity is present in skeletal muscle [1]. Its indispensable member, myeloid differentiation factor (MyD) 88 [2–3], interacts with TLR4 to initiate nuclear factor-κB (NF-κB), which then up-regulates the expression of the genes encoding pro-inflammatory cytokines during LPS challenge. As well as TLR4, nucleotide-binding oligomerization domain protein (NOD) 1 and NOD 2 can activate NF-κB to release pro-inflammatory cytokines [4–6]. The up-regulation of the pro-inflammatory cytokines can cause loss of lean body mass [7–9]. In addition, pro-inflammatory cytokines can lead to muscle atrophy partially via changing the Akt / Forkhead Box O (FOXO)/ubiquitin-proteasome proteolysis (UPP) pathway [10]. Akt/FOXO signaling cascade is an important signaling mechanism in the prosurvival pathway [11]). UPP is the primary proteolytic system, which can degrade most cell proteins [12] and contribute to 75% protein degradation during skeletal muscle atrophy [13–14]. Therefore, UPP serves a critical function in controlling levels of specific proteins. Moreover, many evidences have shown that mammalian target of rapamycin (mTOR) pathway also plays a very crucial role on protein synthesis.

Recent findings have revealed nonessential amino acids in diet are also crucial for animal’s growth and health [15]. Glutamate (Glu) is one of the nonessential amino acids, however, evidence has shown that Glu is a conditionally essential amino acid for weaning piglets. Glu supplementation (as in monosodium glutamate) in postweaning piglet’s diets can be up to 4% without any side-effects, even improve growth performance of piglets for 21d [16]. Nagata reported that a high intake of Glu significantly decreased the risk of total stroke mortality in women [17]. In addition, López-Colomé et al. reported that Glu activated protein synthesis at transcriptional and translational levels in retina [18]. Wu found that Glu was a functional amino acid participating in many kinds of metabolism pathways, including protein production [19].

Skeletal muscle is an important site to regulate the whole body metabolism. Lipopolysaccharide (LPS) challenge can decrease protein synthesis and increase protein degradation in muscle [20], and further induce muscle protein loss or muscle atrophy. However, whether Glu has an effect on the protein loss in the muscle after LPS challenge has not been systematically investigated. Therefore, in this study, we targeted to investigate the effect of Glu inclusion in the weaning piglet diet on muscle protein loss via TLR4, NODs, Akt/FOXO/ and mTOR signaling pathways in skeletal muscle after LPS challenge.

## Materials and methods

### Experimental design

Twenty four healthy weaned piglets (Duroc × Large White × Landrace, 7.02 ± 0.21 kg) were purchased from Hubei Oden Agriculture and Animal Husbandry Technology Co., Ltd and randomly assigned into four treatments: (1) Control [control diet (0% Glu) and saline injection]; (2) LPS + 0% Glu [control diet and LPS (*Escherichia coli* serotype 055: B5; Sigma Chemical, St. Louis, MO) challenge]; (3) LPS + 1.0% Glu (1.0% Glu-supplemented diet and LPS challenge); (4) LPS + 2.0% Glu (2.0% Glu-supplemented diet and LPS challenge). Each treatment had six replicates, and each replicate had one piglet. In this experiment, we focused on determining the response to dietary Glu supplementation among LPS-challenged pigs, so we did not include the effect of Glu without LPS challenge. However, previous studies showed that without LPS challenge, dietary supplementation of 1.0% and 2.0% Glu did not affect growth performance (unpublished data). The diets were isonitrogenous with alanine (purity > 99.5%, Wuhan Amino Acid Bio-Chemical Co., Ltd), and the nutrient composition was required according to NRC (1998) [21], which is shown in Table 1. The experiment was lasted for 28 days. On d 28, the piglets in the LPS-challenge groups were injected intraperitoneally with LPS on 100 μg/kg body weight (BW), and the piglets in the control group were injected with the same volume of 0.9% NaCl solution. The L-Glu we used was purchased from Wuhan Amino Acid Bio-Chemical Co., Ltd (purity > 99.1%).

**Table 1:** Ingredient composition of diets (as fed basis).

Items	Concentration
Ingredients	g/kg
Corn	564
Soybean meal (44% CP)	224
Wheat middling	50
Fish meal	36
Soy protein concentrate	14
Fat powder^a^	20
Defatted milk-replacer powder	30
Limestone	9.4
Dicalcium phosphate	12.2
Salt	3.4
Alanine^b^	12.1
Cornstarch^b^	7.9
Acidifier^c^	2.0
L-Lysine. HCl (78.8% Lysine)	2.7
DL-Methionine (99% methionine)	1.0
L-Threonine (98% threonine)	0.8
Butylated hydroquinone	0.5
Vitamin and mineral premix^d^	10
Nutrient composition	g/kg
Digestible energy^e^^,^^f^ (MJ/kg)	13.7
Crude protein (g)	192
Crude fat (g)	47.7
Calcium (g)	8.9
Total phosphorus (g)	6.7
Amino acid concentrations^g^	g/kg
Total aspartate + asparagine	16.5
Total glutamate + glutamine	29.7
Arginine	9.6
Lysine	10.2
Serine	9
Threonine	7.4
Proline	11.1
Glycine	7
Alanine	20
Histidine	5.1
Leucine	14.5
Isoleucine	5.5
Tyrosine	4.2
Phenylalanine	7.1
Valine	6.3

^a^ A rumen-stable fat powder, purchased from Berg + Schmidt, German.

^b^ In the 1.0% glutamate diet, 1.21% alanine and 0.79% cornstarch were replaced by 1.0% glutamate, 0.61% alanine and 0.39% cornstarch. In the 2.0% glutamate diet, 1.21% alanine and 0.79% cornstarch was replaced by 2.0% glutamate. All diets were isonitrogenous.

^c^ A compound acidifier including lactic acid and phosphoric acid, provided by Wuhan Fanhua Biotechnology Company, Wuhan, China.

^d^ The vitamin and mineral premix (defatted rice bran as carrier) provided the following amounts per kilogram of complete diet: retinol acetate, 2700 μg; cholecalciferol, 62.5 μg; dl-α-tocopheryl acetate, 20 mg; menadione, 3 mg; vitamin B_12_, 18 μg; riboflavin, 4 mg; niacin, 40 mg; pantothenic acid, 15 mg; choline chloride, 400 mg; folic acid, 700 μg; thiamin, 1.5 mg; pyridoxine, 3 mg; biotin, 100 μg; Zn, 80 mg (ZnSO_4_·7H2O); Mn, 20 mg (MnSO_4_·5H_2_O); Fe, 83 mg (FeSO_4_·H_2_O); Cu, 25 mg (CuSO_4_·5H_2_O); I, 0.48 mg (KI); Se, 0.36 mg (Na_2_SeO_3_·5H_2_O).

^e^ Based on diets containing cornstarch.

^f^ Calculated.

^g^ Analyzed.

### Animal feeding

The animal use protocol for this research was approved by the Animal Care and Use Committee of Wuhan Polytechnic University. All piglets were housed in a stainless pens with 1.20 × 1.10 m^2^, and the temperature was 25~27°C. During the whole experimental period, the tested diets and water were provided *ad libitum*.

### Muscle samples collection

In our previous studies, we found that tissue was damaged at 4 h after LPS injection [22–23]. Therefore, on day 28, all the pigs were slaughtered under anesthesia with an intravenous injection of sodium pentobarbital (80 mg/kg BW) at 4 h after LPS or saline injection. Then the gastrocnemius muscle was harvested under the semitendinosus and biceps flexor cruris (left hind leg), and the longissimus dorsi (LD) muscle was collected from the 10th rib (right side of the carcass), respectively. All the muscle samples were frozen in liquid nitrogen immediately, and then stored at -80°C for further analysis.

### Muscle protein, DNA and RNA measurement

Muscle DNA, RNA and protein contents were determined according to the published methods. Briefly, the mucosa (about 0.5g) was homogenized in ice-cold PBS (0·05M-Na_3_PO_4_, 2·0M NaCl, 2 ×10^−3^ M-EDTA, pH 7·4). Mucosal protein content was measured using a detergent-compatible protein assay (Bio-Rad Laboratories, Hercules, CA, USA) [24]. DNA content was assayed by a fluorometric method [25]. RNA levels was determined by ultraviolet absorption at 232·nm and 260·nm [26].

### Muscle mRNA expression analysis

The genes expression, including TLR4, MyD88, IL-1 receptor-associated kinase (IRAK) 1, TNF receptor-associated factor (TRAF) 6, NOD1, NOD2, receptor-interacting serine/threonine-protein kinase (RIPK) 2, NF-κB, TNF-α (TLR4 and NOD signaling pathways), Akt1, FOXO1, FOXO4, muscle atrophy F-box (MAFbx) and muscle RING finger 1 (MuRF1) (Akt/FOXO/UPP signaling pathway) were measured by real-time PCR according to Liu et al [27]. Briefly, total RNA was isolated by the Trizol reagent (#9108, TaKaRa Biotechnology (Dalian) Co., Ltd., Dalian, China), and cDNA synthesis was conducted by PrimeScript^®^ RT reagent kit (#RR047A, TaKaRa Biotechnology (Dalian) Co., Ltd., Dalian, China) according to the manufactures instruction. Quantitative results of the mRNA expression for the target genes was carried out on a ABI 7500 Real-Time PCR System (Applied Biosystems, Life Technologies) using a SYBR^®^ Premix Ex TaqTM (Tli RNaseH Plus) qPCR kit (#RR420A, TaKaRa Biotechnology (Dalian) Co., Ltd., Dalian, China). The PCR cycling conditions were 95°C × 30 s, followed by 40 cycles of 95°C × 5 s and 60°C × 34 s. The primers were designed with Primer 6.0 software and synthesized by TAKARA Biotechnology (Dalian) Co., LTD, China. The primers sequences are shown in Table 2, and the 2^-ΔΔCT^ method was used to calculate the mRNA expression of the target genes relative to GAPDH [28].

**Table 2:** Primer sequences used for real-time PCR.

Gene	Forward (5'—3')	Reverse (5'—3')	Efficiency (%)	Product length (bp)	Accession numbers
TLR4	TCAGTTCTCACCTTCCTCCTG	GTTCATTCCTCACCCAGTCTTC	96	166	GQ503242.1
MyD88	GATGGTAGCGGTTGTCTCTGAT	GATGCTGGGGAACTCTTTCTTC	102	148	AB292176.1
IRAK1	CAAGGCAGGTCAGGTTTCGT	TTCGTGGGGCGTGTAGTGT	96	115	XM_003135490.1
TRAF6	CAAGAGAATACCCAGTCGCACA	ATCCGAGACAAAGGGGAAGAA	101	122	NM_001105286.1
NOD1	CTGTCGTCAACACCGATCCA	CCAGTTGGTGACGCAGCTT	97	57	AB187219.1
NOD2	GAGCGCATCCTCTTAACTTTCG	ACGCTCGTGATCCGTGAAC	99	66	AB195466.1
RIPK2	CAGTGTCCAGTAAATCGCAGTTG	CAGGCTTCCGTCATCTGGTT	103	206	XM_003355027.1
NF-κB	AGTACCCTGAGGCTATAACTCGC	TCCGCAATGGAGGAGAAGTC	100	133	EU399817.1
TNF-α	TCCAATGGCAGAGTGGGTATG	AGCTGGTTGTCTTTCAGCTTCAC	100	67	NM_214022.1
Akt1	GAAGAAGGAGGTCATCGT	GGACAGGTGGAAGAAGAG	98	178	NM_001159776.1
FOXO1	TTCACCAGGCACCATCAT	GAGGAGAGTCGGAAGTAAGT	101	236	NM_214014.2
FOXO4	TGGAGTGTGACATGGATAAC	CTCATCTCTGAAGCAAGGAA	99	122	XM_003135172.3
MAFbx	TCACAGCTCACATCCCTGAG	GACTTGCCGACTCTCTGGAC	98	167	NM_001044588.1
MuRF1	ATGGAGAACCTGGAGAAGCA	ACGGTCCATGATCACCTCAT	103	219	FJ905227.1
GAPDH	CGTCCCTGAGACACGATGGT	GCCTTGACTGTGCCGTGGAAT	100	194	AF017079.1

TLR, toll-like receptor; MyD88, myeloid differentiation factor 88; IRAK1, IL-1 receptor-associated kinase 1; TRAF6, TNF receptor-associated factor 6; NOD, nucleotide-binding oligomerization domain protein; RIPK2, receptor-interacting serine/threonine-protein kinase 2; NF-κB, nuclear factor-κB; TNF-α, tumor necrosis factor-α; FOXO, Forkhead Box O; MAFbx, muscle atrophy F-box; mTOR, mammalian target of rapamycin; MuRF1, muscle RING finger 1.

### Muscle protein expression analysis

Protein expression in muscles was analyzed by Western blot according to our previous studies [29–30]. Briefly, proteins in supernatant fluids (65 μg) were separated on 12% sodium dodecyl sulfate-polyacrylamide gel, and then transferred onto polyvinylidene difluoride membranes for immunoblotting. The first antibodies including total Akt (t-Akt, #9272), phosphorylated Akt (p-Akt, Ser^473^, #9271), total FOXO1 (t-FOXO1, #9454), phosphorylated FOXO1 (p-FOXO1, Ser^256^, #9461), total mTOR (t-mTOR, #2972), phosphorylated mTOR (p-mTOR, Ser^2448^, #2971), total 4EBP1 (t-4EBP1, #9452) and phosphorylated 4EBP1 (p-4EBP1, Thr^70^, #9455) were purchased from Cell Signaling (Danvers, MA), and GAPDH (V-18) (#sc-20357) were purchased from Santa Cruz Biotechnology (Texas, USA). All first antibodies were diluted into 1:1000. The secondary antibodies including goat anti-rabbit IgG-HRP (#ANT020) and rabbit anti-goat IgG-HRP (#ANT021) were purchased from Antgene Biotech (Wuhan, China) and were diluted into 1:5000. The bands were analyzed by densitometry using GeneTools software (Syngene), and the abundance of the phosphorylated proteins was normalized to the total protein contents.

### Statistical analysis

The experimental data were analyzed by ANOVA with SPSS 17.0 software. Differences among treatments were determined by Duncan’s multiple range tests. All data were expressed as means ± SE. The statistical significance level for all analyses was set at p<0.05. A one-way MANOVA was used to determine the effect size (partial eta squared) and the statistical power of the model.

## Results

### Contents of protein, DNA and RNA in muscles

**Fig 1:**
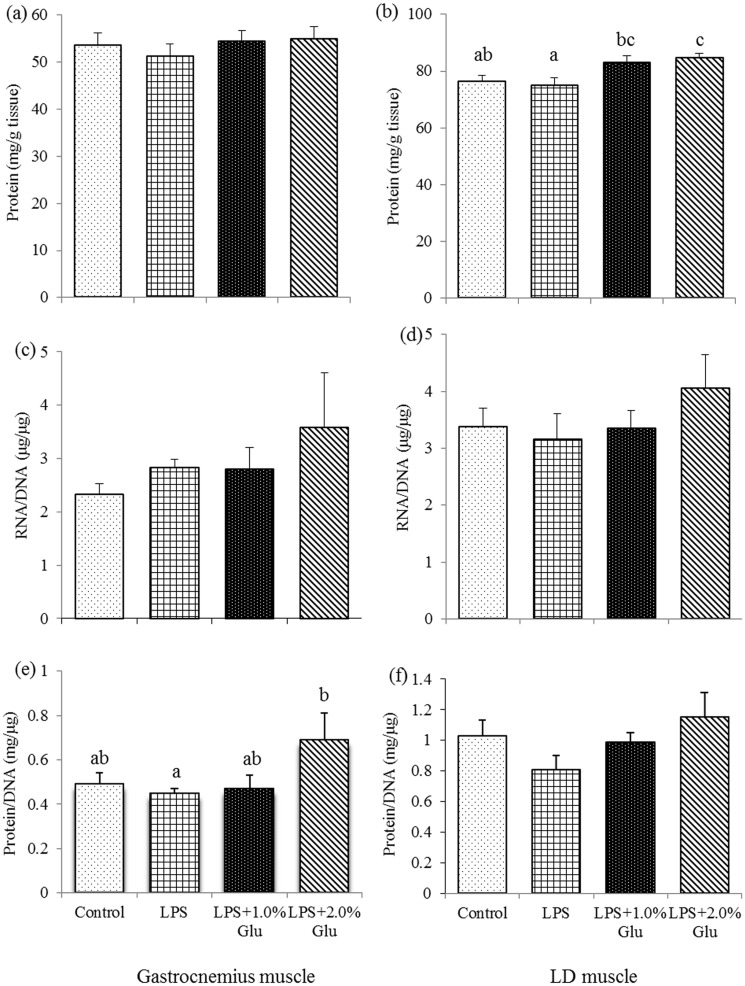
Effects of glutamate (Glu) supplementation on the protein content in gastrocnemius muscle (a) and LD muscle (b), the RNA: DNA ratio in gastrocnemius muscle (c) and LD muscle (d), and the protein: DNA ratio in gastrocnemius muscle (e) and LD muscle (f) in the weaned piglets at 4 h after Escherichia coli lipopolysaccharide (LPS) challenge. Mean ± SE; n = 6, one piglet per pen. ^a,b,c^ Mean values with unlike letters were significantly different (*P*<0.05). A one-way MANOVA revealed the partial eta squared was 0.349, and the power to detect the effect was 0.711. Control (non-challenged control), piglets fed a control diet and injected with sterile saline; LPS (LPS-challenged control), piglets fed the same control diet and injected with LPS; LPS + 1.0% Glu, piglets fed a 1.0% Glu diet and injected with LPS; LPS + 2.0% Glu, piglets fed a 2.0% Glu diet and injected with LPS.

As shown in Fig 1, Glu supplementation increased the protein content (Fig 1b) in LD muscle (*P*<0.05), and the protein/DNA ratio (Fig 1e) in gastrocnemius muscle (*P*<0.05) after LPS challenge.

### Key genes mRNA expression in TLR4 and NOD signaling pathways in muscles

As shown in Table 3, compared to the control group, LPS challenge increased the mRNA expression of TLR4, MyD88, NOD2 and RIPK2 in gastrocnemius muscle, and the mRNA expression of MyD88 in LD muscle (*P*<0.05), and decreased the mRNA expression of NOD1 in gastrocnemius muscle and IRAK1 in LD muscle (*P*<0.05). In addition, as for the LPS-challenged piglets, Glu supplementation up to 2.0% decreased TLR4, IRAK1, RIPK2 and NF-κB mRNA expression in gastrocnemius muscle (*P*<0.05), and MyD88 mRNA expression in LD muscle (P<0.05).

**Table 3:** The effect of glutamate on the key genes mRNA expression in TLR4 and NOD signaling pathways in muscles in LPS-challenged piglets ^1^.

	Treatments			
Item	Control	LPS+0% Glu	LPS+1.0% Glu	LPS+2.0% Glu
Gastrocnemius muscle				
TLR4	1.00±0.16^a^	1.86±0.13^c^	1.65±0.25^b^^c^	1.16±0.20^a^^b^
MyD88	1.00±0.13^a^	1.84±0.16^b^	1.89±0.24^b^	1.41±0.21^a^^b^
IRAK1	1.00±0.15^b^	0.93±0.11^b^	0.81±0.11^a^^b^	0.56±0.10^a^
TRAF6	1.00±0.13	0.99±0.13	0.94±0.12	0.73±0.13
NOD1	1.00±0.12^b^	0.63±0.12^a^	0.71±0.12^a^^b^	0.52±0.09^a^
NOD2	1.00±0.18^a^	2.45±0.32^b^	2.19±0.40^b^	1.66±0.24^a^^b^
RIPK2	1.00±0.12^a^	2.25±0.18^b^	2.05±0.32^b^	1.24±0.19^a^
NF-κB	1.00±0.12^a^^b^	1.31±0.15^b^	1.12±0.14^a^^b^	0.74±0.08^a^
TNF-α	1.00±0.31	1.17±0.31	1.28±0.26	0.86±0.22
LD muscle				
TLR4	1.00±0.17	1.42±0.52	1.68±0.44	0.84±0.19
MyD88	1.00±0.08^a^	1.58±0.21^b^	1.59±0.17^b^	1.06±0.13^a^
IRAK1	1.00±0.59^b^	0.69±0.09^a^	0.72±0.07^a^	0.61±0.08^a^
TRAF6	1.00±0.04	0.94±0.04	0.88±0.09	0.79±0.11
NOD1	1.00±0.12^a^^b^	0.68±0.19^a^^b^	1.16±0.38^b^	0.44±0.09^a^
NOD2	1.00±0.05	1.89±0.42	1.95±0.37	1.40±0.27
RIPK2	1.00±0.02^a^	1.47±0.17^a^^b^	1.85±0.34^b^	1.57±0.24^a^^b^
NF-κB	1.00±0.05	1.08±0.08	1.12±0.11	0.87±0.10
TNF-α	1.00±0.19^b^	0.82±0.17^a^^b^	0.80±0.09^a^^b^	0.51±0.07^a^

TLR4, Toll-like receptor; MyD88, Myeloid differentiation factor 88; IRAK1, IL-1 receptor-associated kinase 1; TRAF6, TNF receptor associated factor 6; NOD1, Nucleotide binding oligomerization domain protein 1; NOD2, Nucleotide binding oligomerization domain protein 2; RIPK2, Receptor-interacting serine/threonine-protein kinase 2; NF-κB, Nuclear factor-κB; TNF-α, Tumor necrosis factor-α. LD, Longissimus dorsi.

^a,b,c^ Mean values with unlike letters were significantly different (*P*<0.05). A one-way MANOVA revealed the partial eta squared was 0.968, and the power to detect the effect was 0.989.

^1^ Values are means ±SE, n = 6 (1 pig/pen).

### Key genes mRNA expression in Akt/FOXO/UPP signaling pathway in muscles

As shown in Table 4, compared to the control group, LPS challenge increased the mRNA expression of FOXO1 and MuRF1 in both gastrocnemius and LD muscles, and decreased the mRNA expression of FOXO4 in LD muscle (*P*<0.05). Glu supplementation up to 2.0% decreased the FOXO1 mRNA expression in LD muscle (*P*<0.05) in LPS-challenged piglets.

**Table 4:** The effect of glutamate on the key genes mRNA expression in Akt/FOXO/UPP signaling pathway in muscles in LPS-challenged piglets ^1^.

	Treatments			
Item	Control	LPS+0% Glu	LPS+1.0% Glu	LPS+2.0% Glu
Gastrocnemius muscle				
Akt1	1.00±0.17	0.67±0.11	0.69±0.07	0.74±0.18
FOXO1	1.00±0.12^a^	1.80±0.24^b^	1.62±0.18^a^^b^	1.57±0.29^a^^b^
FOXO4	1.00±0.10	0.76±0.11	0.84±0.08	0.91±0.14
MAFbx	1.00±0.21	0.88±0.22	1.12±0.32	0.96±0.21
MuRF1	1.00±0.14^a^	2.62±0.40^b^	2.67±0.49^b^	2.93±0.55^b^
LD muscle				
Akt1	1.00±0.09^b^	0.78±0.08^a^^b^	0.86±0.10^a^^b^	0.66±0.09^a^
FOXO1	1.00±0.08^a^	2.81±0.17^c^	2.67±0.29^c^	1.99±0.16^b^
FOXO4	1.00±0.04^b^	0.81±0.06^a^	0.82±0.02^a^	0.67±0.06^a^
MAFbx	1.00±0.15	0.79±0.12	0.87±0.13	0.68±0.09
MuRF1	1.00±0.06^a^	2.90±0.21^b^	3.20±0.15^b^	2.63±0.49^b^

FOXO, Forkhead box O; MAFbx, Muscle atrophy F-box; MuRF1, Muscle ring finger 1; LD, Longissimus dorsi.

^a,b,c^ Mean values with unlike letters were significantly different (*P*<0.05). A one-way MANOVA revealed the partial eta squared was 0.765, and the power to detect the effect was 1.000.

^1^ Values are means ± SE, n = 6 (1 pig/pen).

### Protein expression of t-Akt, p-Akt, t-FOXO1 and p-FOXO1 in muscles

**Fig 2:**
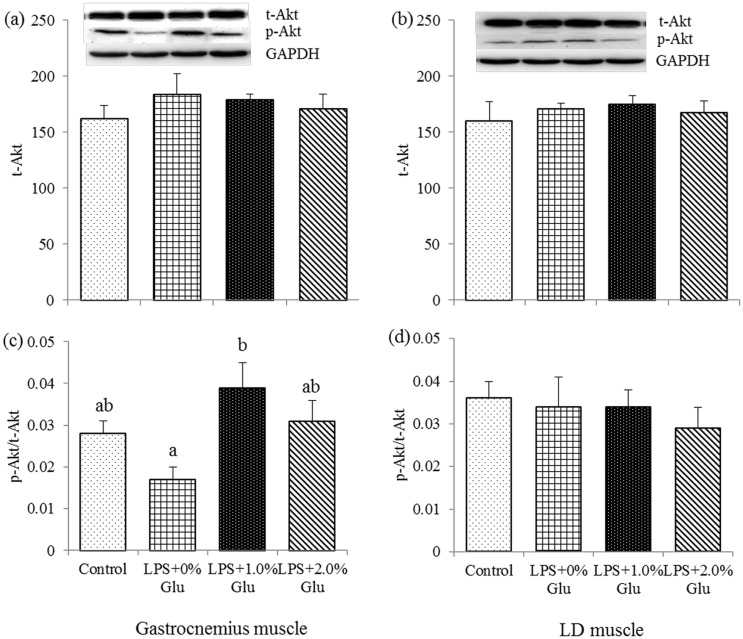
Effects of glutamate (Glu) supplementation on the protein abundance of t-Akt in gastrocnemius muscle (a) and LD muscle (b), and p-Akt: t-Akt ratio in gastrocnemius muscle (c) and LD muscle (d) in the weaned piglets at 4 h after Escherichia coli lipopolysaccharide (LPS) challenge. Mean ± SE; n = 6, one piglet per pen. ^a,b^ Mean values with unlike letters were significantly different (*P*<0.05). A one-way MANOVA revealed the partial eta squared was 0.550, and the power to detect the effect was 0.938. Control (non-challenged control), piglets fed a control diet and injected with sterile saline; LPS (LPS-challenged control), piglets fed the same control diet and injected with LPS; LPS + 1.0% Glu, piglets fed a 1.0% Glu diet and injected with LPS; LPS + 2.0% Glu, piglets fed a 2.0% Glu diet and injected with LPS.

**Fig 3:**
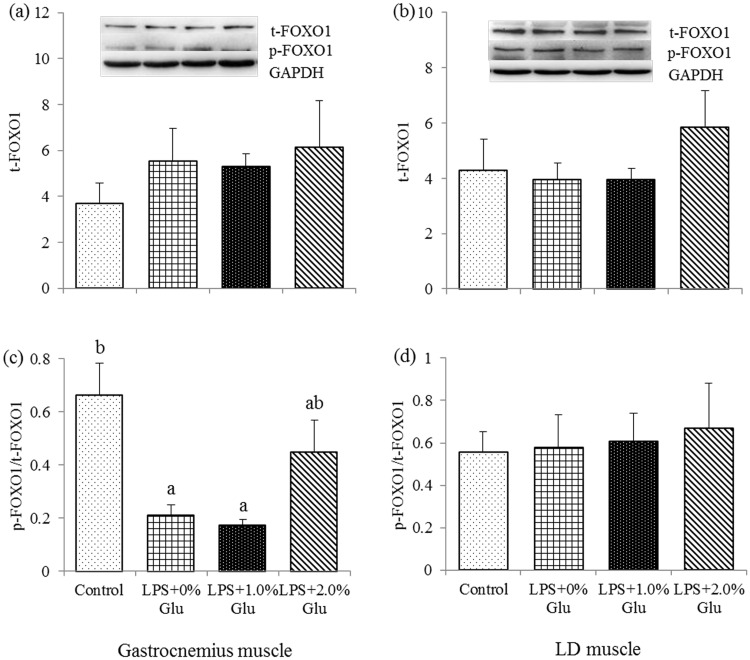
Effects of glutamate (Glu) supplementation on the protein abundance of t-FOXO1 in gastrocnemius muscle (a) and LD muscle (b), and p-FOXO1: t-FOXO1 ratio in gastrocnemius muscle (c) and LD muscle (d) in the weaned piglets at 4 h after Escherichia coli lipopolysaccharide (LPS) challenge. Mean ± SE; n = 6, one piglet per pen. ^a,b^ Mean values with unlike letters were significantly different (*P*<0.05). A one-way MANOVA revealed the partial eta squared was 0.550, and the power to detect the effect was 0.938. Control (non-challenged control), piglets fed a control diet and injected with sterile saline; LPS (LPS-challenged control), piglets fed the same control diet and injected with LPS; LPS + 1.0% Glu, piglets fed a 1.0% Glu diet and injected with LPS; LPS + 2.0% Glu, piglets fed a 2.0% Glu diet and injected with LPS.

As shown in Figs 2 and 3, compared to the control group, LPS challenge decreased p-FOXO1/t-FOXO1 ratio (Fig 3c) in gastrocnemius muscle (*P*<0.05). Glu supplementation up to 1.0% increased p-Akt/t-Akt ratio (Fig 2c) in gastrocnemius muscle after LPS challenge (*P*<0.05).

### Protein expression of t-mTOR, p-mTOR, t-4EBP1 and p-4EBP1 in muscles

**Fig 4:**
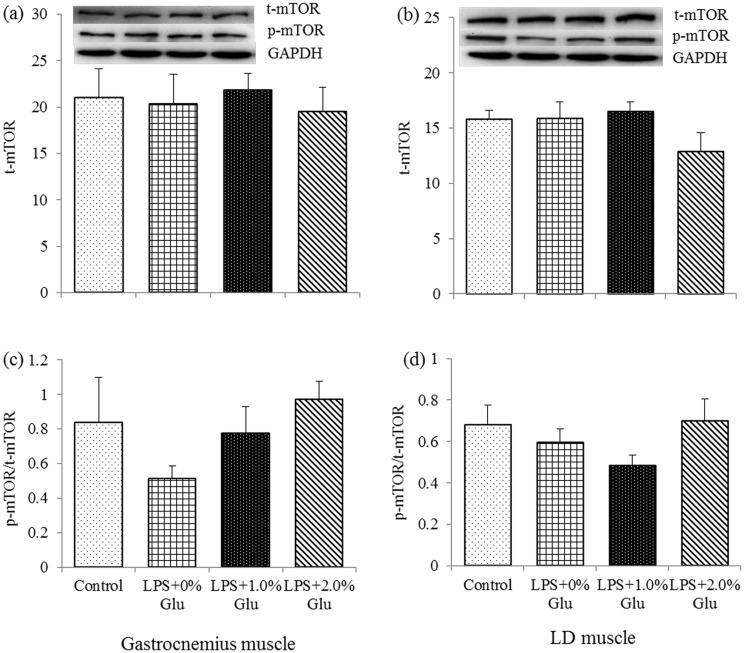
Effects of glutamate (Glu) supplementation on the protein abundance of t-mTOR in gastrocnemius muscle (a) and LD muscle (b), and p-mTOR: t-mTOR ratio in gastrocnemius muscle (c) and LD muscle (d) in the weaned piglets at 4 h after Escherichia coli lipopolysaccharide (LPS) challenge. Mean ± SE; n = 6, one piglet per pen. A one-way MANOVA revealed the partial eta squared was 0.481, and the power to detect the effect was 0.949. CONTR (non-challenged control), piglets fed a control diet and injected with sterile saline; LPS (LPS-challenged control), piglets fed the same control diet and injected with LPS; LPS + 1.0% Glu, piglets fed a 1.0% Glu diet and injected with LPS; LPS + 2.0% Glu, piglets fed a 2.0% Glu diet and injected with LPS.

**Fig 5:**
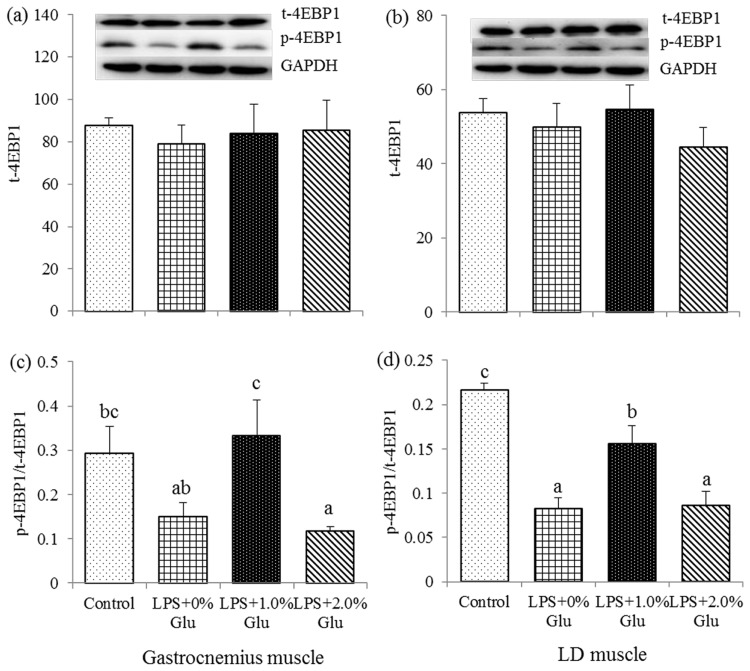
Effects of glutamate (Glu) supplementation on the protein abundance of t-4EBP1 in gastrocnemius muscle (a) and LD muscle (b), and p-4EBP1: t-4EBP1 ratio in gastrocnemius muscle (c) and LD muscle (d) in the weaned piglets at 4 h after Escherichia coli lipopolysaccharide (LPS) challenge. Mean ± SE; n = 6, one piglet per pen. ^a,b,c^ Mean values with unlike letters were significantly different (*P*<0.05). A one-way MANOVA revealed the partial eta squared was 0.481, and the power to detect the effect was 0.949. CONTR (non-challenged control), piglets fed a control diet and injected with sterile saline; LPS (LPS-challenged control), piglets fed the same control diet and injected with LPS; LPS + 1.0% Glu, piglets fed a 1.0% Glu diet and injected with LPS; LPS + 2.0% Glu, piglets fed a 2.0% Glu diet and injected with LPS.

As shown in Fig 4, both LPS challenge and Glu inclusion had no effect on the protein expression of t-mTOR and p-mTOR: t-mTOR ratio (*P*>0.05). As shown in Fig 5, compared to the control group, LPS challenge decreased p-4EBP1/t-4EBP1 in LD muscle (Fig 5c) (*P*<0.05). Glu inclusion up to 1.0% increased the p-4EBP1/t-4EBP1 ratio in both gastrocnemius (Fig 5c) and LD muscles (Fig 5d) after LPS challenge (*P*<0.05).

## Discussion

Skeletal muscle is the most abundant tissue of the body. LPS can trigger skeletal muscle damage [31], mainly characterized by a decrease in total protein [32], accordingly, the damaged muscle has an ability to regenerate new muscle fibers [33], including the increased protein synthesis. Protein/DNA ratio can be a measure of muscle protein concentration [34] and cell size [35]. In the current study, we found Glu inclusion increased protein/DNA ratio in gastrocnemius muscle, as well as protein content in LD muscle after LPS challenge, which indicates that Glu supplementation stimulated protein synthetic capacity and inhibited protein degradation in muscle cells after LPS challenge.

TLR4 play an important role to trigger mammalian innate immune response by recognizing bacterial LPS [36]. TLR4 can transfer signal to MyD88 and IRAK1, which then stimulate NF-κB to produce pro-inflammatory cytokines, such as TNF-α. Inhibiting pro-inflammatory cytokine production by blocking TLR4/NF-κB pathway can alleviate LPS-induced inflammatory responses [37]. As well as TLR4, NOD1 and NOD2 can activate NF-κB to induce the production of pro-inflammatory cytokines [4–6]. RIPK2 is an essential downstream adaptor protein in NODs signaling pathway, which can stimulate NF-κB pathway [38–39]. Moreover, previous study has reported that pro-inflammatory cytokines such as TNF-α promoted the loss of muscle protein in skeletal muscle [40]. In the current study, in consistent with Frisard et al (2015) [41] who found that LPS challenge stimulated TLR4 activation in skeletal muscle, we also found LPS injection up-regulated TLR4 mRNA expression, which further increased MyD88 mRNA expression in gastrocnemius muscle. Glu is an important precursor for glutamine (Gln). Kessel et al (2008) reported that oral Gln decreased the mRNA expression of TLR4 and MyD88, and resultantly alleviated intestinal mucosal injury caused by LPS in rat [42]. In this study, we found Glu inclusion reduced TLR4, IRAK1, and NOD2 mRNA expression in gastrocnemius muscle, and MyD88 mRNA expression in LD muscle after LPS challenge. In addition, Glu inclusion also decreased NF-κB and RIPK2 mRNA expression in gastrocnemius muscle. These results indicates that Glu supplementation had a positive effect on alleviating muscle protein degradation partially by suppressing the mRNA expression of TLR4 and NODs signaling-related genes after LPS challenge.

In addition, LPS interacts with TLR4/MD2 complex to initiate the inflammatory response [36, 43] and leads to muscle atrophy, which might be partially mediated by the Akt/FOXO/UPP pathway [10]. Phosphorylation of Akt inhibits proteolytic transcription factors [44]. Full activity of Akt requires phosphorylation, which stimulates protein synthesis and induces FOXO1 phosphorylating and inactivating to inhibit protein degradation [45]. FOXO initially stimulates protein degradation [46], and participates in MuRF1 and MAFbx transcription during muscle atrophy [45, 47–48]. Both MuRF1 and MAFbx are relied by UPP to degrade specific proteins within the cells. Crossland et al. found that LPS challenge droved Akt1 and FOXO to stimulate muscle protein degradation [10]. In the current study, we found that Glu inclusion decreased FOXO1 mRNA expression in LD muscle, and increased p-Akt/t-Akt ratios in gastrocnemius muscle after LPS challenge, which suggests that Glu alleviated muscle protein degradation through influencing Akt-FOXO signaling pathway, however, its inclusion could have no effect on UPP pathway evidenced by the unchanged MuRF1 and MAFbx mRNA expression.

Amino acids regulate protein synthesis and cell growth mainly through the mTOR signaling pathway [49–50]. Glu, as an efficient energy resource for the gut of animals, is also an important precursor for arginine and glutamine, which have been reported to stimulate mTOR and eukaryotic initiation factor (eIF) 4E-binding protein-1 (4EBP1) phosphorylation to increase protein synthesis [51]. LPS challenge could impair muscle protein synthesis partially resulting from the reduced activity of mTOR kinase, which was supported by the decreased phosphorylation of 4EBP1 [52–53]. 4EBP1 is one of the downstream targets in mTOR signaling pathway, which is mainly to control the rate of protein synthesis [54]. In addition, the Akt-dependent signaling pathway can inhibit proteolysis and stimulate protein synthesis in muscle via activating 4EBP1 [55]. The excitatory amino acid transporters 3 (EAAT3), which was the glutamate transporter, exists in many tissues, including skeletal muscle [56]. Almilaji et al. reported that EAAT3 could be powerfully up-regulated by mTOR, the later could augment carrier protein concentration in the cell membrane [57]. Therefore, the increased EAAT3 induced by the mTOR could theoretically augment Glu transposition. In the present study, we found Glu supplementation increased p-4EBP1/t-4EBP1 ratio both in gastrocnemius muscle and LD muscle after LPS challenge, which indicates that Glu supplementation could stimulate protein synthesis via activating mTOR signaling pathway, not only by increasing 4EBP1 phosphorylation, but probably by up-regulating Glu transposition.

## Conclusion

The results from this study showed that Glu supplementation alleviated muscle protein degradation after LPS challenge not only by suppressing the mRNA expression of TLR4 and NODs signaling-related genes, but by modulating Akt-FOXO signaling pathway to decrease protein degradation and activating mTOR signaling pathway to increase protein synthesis. These results indicated that Glu supplementation might be an effective strategy to alleviate LPS-induced protein loss in muscle after LPS challenge.
